# Exploring links between sensory processing sensitivity, professional quality of life, and negative affectivity in clinical and counselling psychologists

**DOI:** 10.1371/journal.pmen.0000362

**Published:** 2025-07-07

**Authors:** Amanda M. McQuarrie, Lorna Susan Jakobson

**Affiliations:** Department of Psychology, University of Manitoba, Winnipeg, Manitoba, Canada; University of Sunderland, UNITED KINGDOM OF GREAT BRITAIN AND NORTHERN IRELAND

## Abstract

Mental health professionals, like professionals in other fields, experience a high risk of burnout. Situational as well as individual factors can increase this risk. The current study explored possible mediators of the relationships between the personality trait of sensory processing sensitivity (SPS) and the risk of poor professional quality of life and negative affectivity in a group of 95 clinical or counselling psychologists. Participants completed self-report measures of SPS, three proposed mediators (perceived negative impacts of the COVID-19 pandemic, compassion satisfaction, and self-compassion) and five different outcome variables (burnout, secondary traumatic stress, stress, anxiety, and depression). Subscale scores on the measures of SPS were used to compute composite scores reflecting the strength of positive (SPS+) and negative (SPS-) trait clusters. The three proposed mediators were entered into each mediation model in parallel to allow us to tease apart the unique roles they played in explaining links between these two facets of SPS and our outcome variables. Reporting higher levels of SPS- traits predicted greater perceived pandemic burden and lower levels of self-compassion and compassion satisfaction. Links between SPS- traits and increased risk of all five negative outcomes were fully or partially mediated by at least two of the mediators. In contrast, high scores on the SPS+ trait cluster predicted higher levels of compassion satisfaction which predicted lower levels of burnout and secondary traumatic stress. This study identified several important risk and protective factors for clinical and counselling psychologists with SPS. By exploring the nature of the association between SPS and professional quality of life and mental health, the findings may inform proactive measures to support those with SPS who work in this vital area.

## Introduction

In recent years, those working in helping professions have been at an increasingly high risk for employment-related burnout and trauma-related employment stress—including vicarious trauma, secondary traumatic stress, and compassion fatigue [1]. These interrelated phenomena can negatively impact organizations [2]. They also impact individuals and are associated with cognitive and emotional problems including feelings of stress, anxiety, and depression, as well as increased physical health concerns and behavioural disturbances [3]. Ultimately, these organizational and personal impacts may also compromise the quality of care that helping professionals are able to offer to people they serve.

Learning more about factors that contribute to negative outcomes in helping professionals is important if we are to properly support these individuals and minimize attrition—particularly during or after events that caused significant disruption, damage, and distress to communities, such as the recent Coronavirus disease (COVID-19) pandemic [4]. Findings from a meta-analysis carried out before the pandemic identified a range of risk factors, such as high caseloads and receiving a lack of support from supervisors, colleagues, family and friends [5]. Edú-Valsania et al. [3] argued that individual factors such as possessing poor coping strategies or certain personality traits can also increase one’s risk. There are commonalities in the mental health outcomes of health workers across the multiple pandemics/epidemics that have occurred in this century (e.g., severe acute respiratory syndrome in 2002, swine flu in 2009, Middle East respiratory syndrome in 2012, and Ebola virus disease in 2014), indicating the continued importance of studying impacts from these pandemics to allow for proper systems-level changes that will help protect workers in the future [4].

One personality trait that has been a focus of research into professional quality of life in recent years is sensory processing sensitivity (SPS) [6]. Approximately one third of the general population possess high levels of this trait [7]. Individuals who strongly express SPS traits are more sensitive to their internal and external environment, process stimuli more deeply, and are more emotionally reactive [8,9] and empathic [10] than those scoring low on this trait. In the model of SPS proposed by Aron and colleagues [8], which was developed within the framework of evolutionary biology, SPS is viewed as a negative-frequency dependent trait—one that confers advantages such as those described above when it occurs in a minority of the population. However, they also stress the importance of having an optimal level of arousal in order to successfully deploy a “sensitive strategy” and that overstimulation can interfere with both cognitive and emotional functioning. This may explain why workers across numerous fields who report high levels of SPS have been found to be at an increased risk of experiencing burnout [6,11–16] in part because they feel things more strongly and are more easily overstimulated [9], and why stronger expression of SPS has also been associated with stronger symptoms of stress, anxiety, and depression in numerous studies [17,18]. The majority of individuals possessing high levels of this trait have most likely developed strategies for general coping and emotion regulation that allow them to function in their typical environment [9]. However, during times of increased global stress these strategies may not be enough, and the risk of poor professional quality of life and negative affectivity may substantially increase.

In the present study, we sought to identify factors that might explain the link between SPS and these outcomes in a sample of clinical and counselling psychologists. A novel aspect of our research is that, informed by recent findings [19,20], we took into account the fact that there are two distinct clusters of SPS traits: a positive cluster (SPS+) that includes sensitivity to subtle stimuli, comfortable or pleasurable sensations, and social-affective and aesthetic cues; and a negative cluster (SPS-) that includes heightened emotional and physiological reactivity and sensitivity to stimuli that make one feel uncomfortable. As SPS- traits have previously been shown to be more strongly related than SPS+ traits to neuroticism and negative clinical outcomes in the general population [21], we expected that the strong expression of these maladaptive SPS- traits would be associated with higher risk of burnout, secondary traumatic stress, and negative affectivity in our sample. This prediction aligns with results from recent work suggesting that traits belonging to the SPS- cluster predict more severe burnout [6] and intensify the impact of job demands on emotional exhaustion [16]. In contrast, we predicted that strong expression of the SPS+ cluster might offer some protection against poor outcomes. This would be consistent with Golonka and Gulla’s [6] finding that sensitivity to subtle stimuli (an SPS+ trait) is linked to less severe burnout and exhaustion.

Another novel aspect of the current study is that it explored three potential mediators of the links between different facets of SPS and our outcome variables. The first potential mediator was the degree to which participants perceived that the COVID-19 pandemic had negatively impacted them. COVID-19 emerged at the end of 2019, with the World Health Organization declaring the spread of this disease a pandemic in March 2020 [22]. Our data were gathered approximately 2.5 years later, when the risk of infection was still high. Health concerns over contracting the virus, financial impacts, worry about family and friends, and various other stressors heavily impacted the mental health of people in Canada and around the world, with one study finding that 38.2% of participants felt that they had experienced a decrease in their mental health compared to pre-pandemic levels [23]. Psychologists and other mental health professionals were not immune to these effects, reporting higher rates of anxiety and depression compared to before the pandemic [24]. This likely partially reflects the fact that these professionals had the added burden of helping clients work through pandemic-related experiences similar to those they themselves were experiencing [25]. Additionally, Kercher and Gossage [24] note that during the pandemic psychologists reported increases in caseloads, responsibilities, and work-related stressors, without concomitant increases in professional or personal support; see also [26,27]. These factors, along with increased psychological distress, supervision frequency, and years in practice, all predicted increased feelings of burnout and secondary traumatic stress in their sample. The idea that pandemic-related burden might mediate the link between SPS- traits and adverse outcomes gains preliminary support from research showing that negative impacts of the pandemic partially mediated the link between SPS and internalizing symptoms in adolescents [28].

A second potential mediator we considered was compassion satisfaction, or the feeling of satisfaction that the mental health professionals in our sample got from their jobs. Compassion satisfaction is a factor that enhances professional quality of life [29]. A prior study investigating the relationship between SPS and professional quality of life in dentists found that components of the SPS- trait cluster [19,20] were negatively associated with compassion satisfaction [13]. This association makes sense, as being more emotionally/physiologically reactive and sensitive to uncomfortable stimuli might lead helping professionals to feel overwhelmed in stressful situations, reducing their ability to take satisfaction from their jobs. In contrast, Meyerson and colleagues [13] found that one component of the SPS+ trait cluster (i.e., aesthetic sensitivity) was positively associated with compassion satisfaction. It is possible that this and other traits in the SPS+ cluster (e.g., sensitivity to sensory comfort/pleasure and social-affective cues) not only offer mental health professionals who strongly express them some protection from experiencing adverse outcomes but also enhance the pleasure they take from helping others. However, Kercher et al. [27] recently reported that, although 90% of their sample of registered psychologists expressed high compassion satisfaction and had no intention of leaving the profession, the risk of experiencing burnout and secondary traumatic stress was still elevated in those reporting more pandemic-related stress and psychological distress.

A third potential mediator that we investigated was self-compassion. Self-compassion refers to a way of relating to ourselves with compassion in times of struggle [30]. Being able to respond to situations with self-compassion is seen as a protective factor for burnout and secondary traumatic stress and is positively related to compassion satisfaction and job satisfaction [31,32], as well as overall well-being [33]. This is important as taking care of oneself enhances one’s ability to care effectively for others. Self-compassion has also been linked to resilience and reduced levels of depression and anxiety during the COVID-19 pandemic [34]. To date, no research we are aware of has explored relationships between SPS and self-compassion directly; however, SPS has been associated with low levels of dispositional mindfulness [35] and those who score low on mindfulness have been found by others to also score low on self-compassion [31]. Indeed, in a widely used self-report measure of this construct, the Self-Compassion Scale [36], mindfulness is considered a component of self-compassion. These findings might lead one to expect that individuals with higher levels of SPS would generally experience lower self-compassion, but we would predict that this negative relationship is primarily driven by SPS- traits. It is important to evaluate this possibility, and the idea that scoring high on SPS+ traits would predict stronger self-compassion and offer protection from burnout.

In light of the research reviewed above, the purpose of the present study was to explore the possible mediating roles of perceived COVID-19 impacts, compassion satisfaction, and self-compassion on the relationships between different facets of SPS and measures of burnout, secondary traumatic stress, and negative affectivity in a sample of clinical and counselling psychologists. We expected that perceived COVID-19 impacts would positively mediate the relationship between SPS- traits and our outcome variables. Thus, scoring high on SPS- traits was expected to predict feeling more impacted by the pandemic, which would predict more adverse outcomes. SPS- and SPS+ traits were expected to relate to compassion satisfaction and self-compassion in different ways. SPS- traits were generally expected to predict lower scores on these variables, and this was expected to increase the risk of adverse outcomes. In contrast, SPS+ traits were expected to predict higher scores on these variables, potentially offering some protection against adverse outcomes.

## Materials and methods

### Participants

We recruited 124 individuals, which exceeded the sample size estimated using G*Power 3.1 [37] needed to achieve a medium effect size with power of.80 and an alpha of.05 for the planned mediation analyses. After data cleaning (described below), however, the final sample included 95 participants, leaving the study slightly underpowered. All were practitioners in clinical or counselling psychology who were currently practicing with at least one client per week in either an assessment or therapy setting and were fluent in English. Participants were recruited through provincial clinical and counselling psychology societies and associations throughout Canada, as well as through the Canadian Psychological Association (the largest national association in the country for the science, practice, and education of psychology). Data were collected between August 1 and October 31, 2022, during which time the majority of restrictions due to COVID-19 were being lifted across Canada after 2.5 years, but the risk of contracting COVID-19 was still high [38].

### General procedure

Participants completed an online survey that included measures tapping into SPS; secondary traumatic stress, compassion satisfaction, and burnout; self-compassion; current symptoms of depression, anxiety, and stress; and impacts of the COVID-19 pandemic. Items from a short attention check measure were randomly distributed throughout the survey. Prior to beginning the survey, prospective participants read an online consent form. Those who responded “yes” to a question at the end of the form asking if they agreed to take part were included in the final sample. Participants were offered the chance to enter a draw for a $50 gift card after completion of the study. The procedure was approved by the university research ethics board.

### Measures

#### Sensory processing sensitivity.

The Highly Sensitive Person Scale (HSPS) [8] is a 27-item self-report measure of SPS. Items comprising this measure are answered using a seven-point Likert scale ranging from 1 (*Not at all*) to 7 (*Extremely*). Responses to relevant items are averaged to produce three subscale scores [21]: low sensory threshold (six items; e.g., “are you bothered by intense stimuli, like loud noises or chaotic scenes”), ease of excitation (12 items; e.g., “do you startle easily”), and aesthetic sensitivity (seven items; e.g., “do you have a rich, complex inner life”). Aron and colleagues [9] recommended that the Orienting Sensitivity subscale of the Adult Temperament Questionnaire (OS) [39] be administered alongside the HSPS to more fully measure the construct of SPS. The OS includes 15 items that are rated on a seven-point Likert scale ranging from 1 (*extremely untrue*) to 7 (*extremely true*). Again, relevant responses are averaged to produce three subscale scores, each with five items: affective perceptual sensitivity (e.g., “I am often consciously aware of how the weather seems to affect my mood”), associative sensitivity (“when I am resting with my eyes closed, I sometimes see visual images”), and neutral perceptual sensitivity (e.g., “I often notice visual details in the environment”).

As noted in the Introduction, recent work has indicated that SPS has both positive (adaptive) and negative (maladaptive) aspects [19,20]. In the present study, the average of the three subscales of the OS and the aesthetic sensitivity subscale of the HSPS was computed to capture the SPS+ trait cluster, and the average of the low sensory threshold and ease of excitation subscales of the HSPS was computed to capture the SPS- trait cluster [20]. Internal consistency was good for both the SPS+ (*α* = .81) and SPS- (*α* = .90) composite scores.

#### Professional quality of life scale.

The Professional Quality of Life Scale (ProQOL-5) [29] is a 30-item self-report measure of the positive and negative feelings one has related to working in a helping profession. Items are answered using a five-point Likert scale ranging from 1 (*never*) to 5 (*very often*). The ProQOL-5 is comprised of three subscales tapping into key features of one’s professional quality of life: Burnout (BO; 10 items; “I feel worn out because of my work as a [helper]”); Secondary Traumatic Stress (STS; 10 items; “I feel as though I am experiencing the trauma of someone I have [helped]”); and Compassion Satisfaction (CS; 10 items; “I feel invigorated after working with those I [help]”). After reverse scoring where indicated, subscales are calculated by finding the sum of responses to relevant items. In the present study, the internal consistency of the BO (*α* = .77), STS (*α* = .84) and CS (*α* = .89) subscales of the ProQOL-5 was considered acceptable to good.

#### Depression anxiety stress scale.

The Depression Anxiety Stress Scale (DASS-21) [40] is a 21-item scale that measures symptoms of depression, anxiety, and stress over the past week. Items are answered using a four-point Likert scale ranging from 0 (*Did not apply to me at all*) to 3 (*Applied to me very much, or most of the time*). Scores on each of the three 7-item subscales are calculated by summing responses to relevant items measuring participants’ levels of stress (e.g., “I tended to over-react to situations”), anxiety (e.g., “I felt I was close to panic”), or depression (e.g., “I felt down-hearted and blue”) and multiplying the sum by two. Higher scores are indicative of greater symptom severity. In the present study, the internal consistency of the Depression (*α* = .91), Anxiety (*α* = .83) and Stress (*α* = .91) subscales of the DASS-21 were considered good.

#### Coronavirus impact scale.

The Coronavirus Impact Scale (CIS) [41] is a 12-item self-report measure used to measure the burden the pandemic placed on individuals across multiple domains. Four items are used to gather descriptive information about diagnoses and severity of COVID-19 infection in oneself and one’s family and friends and are not included in the total score. The remaining eight items assess the negative impact of the COVID-19 pandemic on routines, family income/employment, food access, access to medical care, access to treatment for mental health problems, access to extended family and non-family social supports, experiences of stress related to pandemic itself, and stress and discord in the family. Responses on these items are entered on a scale from 0 (*none*) to 3 (*severe*) and are summed to provide an overall measure of the perceived burden of the pandemic. The internal consistency of this measure in the current study was acceptable (*α* = .79).

#### Self-compassion scale.

The Self-Compassion Scale (SCS) [36] is a 26-item scale that measures the frequency of one’s positive and negative thoughts and feelings towards themselves during challenging times. Items are answered using a five-point Likert scale ranging from 1 (*Almost never*) to 5 (*Almost always*). The SCS is comprised of six subscales. Three tap into key features of self-compassion: self-kindness (five items; “I try to be loving toward myself when I’m feeling emotional pain”); common humanity (four items: “When things are going badly for me, I see the difficulties as part of life that everyone goes through”); and mindfulness (four items: “When something upsets me, I try to keep my emotions in balance”). The three remaining subscales measure uncompassionate responses towards the self: self-judgement (five items; “I’m disapproving and judgmental about my own flaws and inadequacies”); isolation (four items; “When I think about my inadequacies, it tends to make me feel more separate and cut off from the rest of the world”); and over-identification (four items; “When I’m feeling down, I tend to obsess and fixate on everything that’s wrong”). The items comprising these three subscales are reverse scored and then a total self-compassion score is calculated by finding the mean of all 26 items. In the current study, the internal consistency of the SCS total score was excellent (*α* = .95).

#### Attention check: The conscientious responders scale.

The five items that comprise the Conscientious Responders Scale (CRS) [42] stipulate how participants should respond (e.g., “To answer this question, please choose option 4 ‘moderately agree’”). The authors stipulate that making more than two errors on these items provides evidence of careless responding.

### Analyses

Data cleaning resulted in the exclusion of 29 participants who did not complete at least one subscale of a measure or who responded incorrectly to more than two attention-check items. Items were checked to ensure proper reverse-scoring and coding, and scores were checked for outliers, linearity, and possible violations to normality. Little’s Missing Completely at Random test was run and confirmed that the missing data were missing completely at random. An Estimation-Maximization algorithm was used to input missing values. Statistical analyses were conducted using IBM SPSS Statistics for Microsoft, Version 30.

Zero-order correlations between the variables of interest were calculated and tested for significance. The Benjamini-Hochberg procedure was used with a False Discovery Rate of 0.05 to reduce the risk of Type 1 errors when testing multiple correlations.

**Fig 1:**
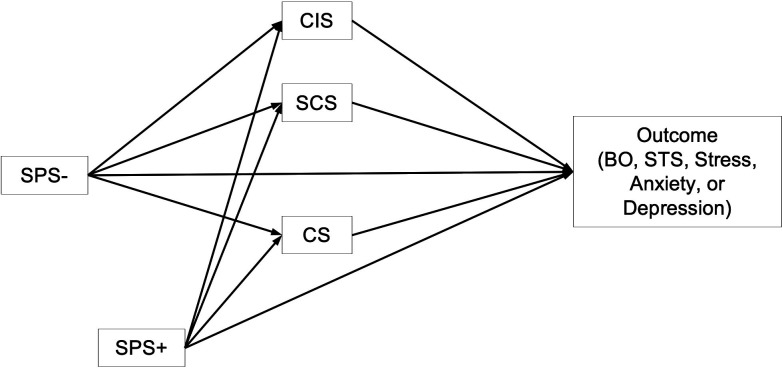
General form of the model used to test for mediating effects on the relationship between facets of SPS and various outcomes. SPS− and SPS+ are the negative and positive trait clusters associated with sensory processing sensitivity, respectively. SPS- is entered as a predictor, SPS+ as a covariate. The mediating variables included the Coronavirus Impact Scale (CIS) total score, the Compassion Satisfaction (CS) subscale of the Professional Quality of Life Scale, and the Self-Compassion Scale (SCS) total score. Outcomes used in various models included measures of Burnout (BO) and Secondary Traumatic Stress (STS) from the Professional Quality of Life Scale, as well as the stress, anxiety, and depression subscales from the Depression Anxiety Stress Scale.

Separate parallel mediation analyses were then run using model 4 in the PROCESS macro [43], to determine if links between the SPS composite scores and our five outcome variables (BO, STS, Stress, Anxiety, and Depression) were mediated by the degree to which participants were negatively impacted by the COVID-19 pandemic, or by their self-reported levels of compassion satisfaction and self-compassion. As shown in Fig 1, the SPS- composite score was entered as the predictor variable and the SPS+ composite score as a covariate. This approach was taken because, generally speaking, the SPS- score was more strongly correlated with the proposed mediators and the outcome variables than the SPS+ score (see Table 1) and was predicted to be the main driver of adverse outcomes. For each mediation analysis, bias-corrected accelerated 95% confidence intervals (CIs) were calculated using 5000 bootstrapped samples.

**Table 1:** Descriptive statistics and Pearson correlation coefficients measuring relationships between variables.

	Sum/Mean (SD)	SPS-	SPS+	CIS	CS	SCS	BO	STS	Stress	Anxiety
SPS-	3.73 (1.13)	--								
SPS+	4.70 (0.70)	0.41^**^	--							
CIS	7.76 (3.70)	0.45^**^	0.28^**^	--						
CS	40.13 (5.59)	-0.19	0.12	-0.25^*^	--					
SCS	3.39 (0.72)	-0.41^**^	-0.21^*^	-0.41^**^	0.37^**^	--				
BO	21.01 (5.40)	0.42^**^	0.08	0.53^**^	-0.69^**^	-0.54^**^	--			
STS	17.95 (5.74)	0.57^**^	0.33^**^	0.56^**^	-0.39^**^	-0.42^**^	0.66^**^	--		
Stress	12.42 (10.03)	0.57^**^	0.31^**^	0.50^**^	-0.20	-0.58^**^	0.57^**^	0.58^**^	--	
Anxiety	4.17 (6.10)	0.52^**^	0.34^**^	0.43^**^	-0.07	-0.38^**^	0.46^**^	0.54^**^	0.73^**^	--
Depression	7.41 (8.83)	0.38^**^	0.30^**^	0.61^**^	-0.35^**^	-0.50^**^	0.63^**^	0.45^**^	0.71^**^	0.64^**^
			Large	Medium	Small	Small	Medium	Large		
			Negative correlations			Positive correlations				

Correlation coefficients are colour-coded based on effect size as indicated above. SPS+ and SPS-, the positive and negative trait clusters associated with sensory processing sensitivity. CIS, Coronavirus Impact Scale. Subscales of the Professional Quality of Life Scale: CS, Compassion Satisfaction; BO, Burnout; STS, Secondary Traumatic Stress. Subscales of the Depression, Anxiety and Stress Scale: Stress, Anxiety, and Depression. SCS, Self-Compassion Scale.

*p < .05

**p < .01

## Results

### Descriptive statistics and zero-order correlations between self-report variables

Descriptive statistics and Pearson correlation coefficients are shown in Table 1. Of particular interest to the present study, the intercorrelations between the three proposed mediators were significant, with CIS being negatively correlated with both CS and SCS, and the latter two variables being positively correlated with one another. Regarding links between the proposed mediators and the predictors, we found that both CIS and SCS scores were correlated with the SPS- and SPS+ scores, although these effects were in opposite directions; thus, those scoring high on either SPS facet reported more pandemic-related stress and lower levels of self-compassion than those scoring low on either SPS facet. Finally, regarding links between the proposed mediators and the outcome variables, we found that: CIS scores were positively correlated with all outcomes; CS scores were negatively correlated with Burnout, Secondary Traumatic Stress, Stress and Depression; and Self-Compassion Scores were negatively correlated with all outcomes.

### Mediation analyses

After controlling for SPS+ traits, reporting stronger SPS- traits positively predicted CIS scores (*b* = 1.31, *p* < .001, 95% Confidence Interval (CI) = [0.65, 1.97]), and negatively predicted CS (*b* = -1.41, *p *= .011, 95% CI = [-2.48, -0.33]) and SCS scores (*b* = -0.25, *p* < .001, 95% CI = [-0.38, -0.12]). The mediation models are shown in Fig 1 and Table 2. Full positive mediation via all three mediators was supported in the model predicting burnout. Partial positive mediation via CIS and CS was supported in the model predicting secondary traumatic stress; and partial positive mediation via CIS and SCS was supported in the models predicting stress and anxiety. Finally, full positive mediation via CIS and SCS was supported in the model predicting depression. These findings suggest that, when controlling for SPS+ traits, strong expression of SPS- traits increased the risk for all negative outcomes by heightening the perceived negative impacts of the pandemic and/or by lowering feelings of compassion satisfaction and/or self-compassion.

**Table 2:** Summary of analyses investigating mediation of the relationships between SPS composite scores and adverse outcomes.

DV	M	Effect of M on DV	Direct effect	Indirect effect	Total effect
		b [95% CI]	c’ [95% CI]	ab [boot 95% CI]	c [95% CI]
SPS- as predictor; SPS + as covariate					
BO	CIS	**0.39 [0.17, 0.60]**	0.64 [-0.10, 1.37]	**0.51 [0.13, 1.05]**	**2.20 [1.22, 3.18]**
	SCS	**-1.37 [-2.49, -0.25]**		**0.34 [0.01, 0.69]**	
	CS	**-0.50 [-0.64, -0.37]**		**0.71 [0.20, 1.35]**	
STS	CIS	**0.47 [0.20, 0.75]**	**1.56 [0.63, 2.48]**	**0.62 [0.11, 1.32]**	**2.64 [1.69, 3.58]**
	SCS	-0.38 [-1.80, 1.03]		0.10 [-0.35, 0.46]	
	CS	**-0.26 [-0.43, -0.09]**		**0.37 [0.06, 0.89]**	
Stress	CIS	**0.56 [0.09, 1.04]**	**2.74 [1.12, 4.37]**	**0.74 [0.07, 1.64]**	**4.67 [3.02, 6.32]**
	SCS	**-5.25 [-7.73, -2.77]**		**1.30 [0.55, 2.13]** ^a^	
	CS	0.08 [-0.22, 0.38]		-0.11 [-0.53, 0.31]	
Anxiety	CIS	**0.35 [0.02, 0.68]**	**1.74 [0.62, 2.87]**	**0.46 [0.01, 1.13]**	**2.43 [1.40, 3.46]**
	SCS	-1.52 [-3.23, 0.20]		**0.38 [0.03, 0.79]**	
	CS	**0.10 [-0.11, 0.31]**		**-**0.14 [-0.53, 0.17]	
Depression	CIS	**1.02 [0.59, 1.45]**	-0.01 [-1.48, 1.45]	**1.34 [0.49, 2.31]**	**2.42 [0.80, 4.03]**
	SCS	**-2.90 [-5.14, -0.67]**		**0.72 [0.22, 1.33]**	
	CS	-0.27 [-0.54, 0.004]		0.37 [-0.01, 1.09]	
SPS+ as predictor; SPS- as covariate					
BO	CIS	**0.39 [0.17, 0.60]**	-0.17 [-1.28, 0.93]	0.23 [-0.17, 0.84]	-0.84 [-2.43, 0.75]
	SCS	**-1.37 [-2.49, -0.25]**		0.06 [-0.33, 0.44]	
	CS	**-0.50 [-0.64, -0.37]**		**-0.96 [-1.88, -0.04]** ^b^ ^,^ ^c^	
STS	CIS	**0.47 [0.20, 0.75]**	1.09 [-0.30, 2.48]	0.28 [-0.22, 0.98]	0.90 [-0.63, 2.43]
	SCS	-0.38 [-1.80, 1.03]		0.02 [-0.22, 0.24]	
	CS	**-0.26 [-0.43, -0.09]**		**-0.49 [-1.14, -0.002]** ^d^	

Shown are the unstandardized coefficients for the direct, indirect, and total effects, with 95% confidence intervals (CIs) indicated. Percentile bootstrap (boot) CIs for the indirect effects were calculated using 5000 bootstrapped samples. Significant effects are shown in bold. SPS- and SPS + are the negative and positive facets of sensory processing sensitivity, respectively. The dependent variables (DVs) included: (a) the Burnout (BO) and Secondary Traumatic Stress (STS) subscales of the Professional Quality of Life Scale; and (b) the Stress, Anxiety, and Depression subscales of the Depression Anxiety Stress Scale. The mediating variables (M) included total scores for the: (a) Coronavirus Impact Scale (CIS); (b) Compassion Satisfaction (CS) subscale of the Professional Quality of Life Scale; and (c) Self-Compassion Scale (SCS).

^a^Indirect effect larger for SCS than CS; indirect contrast 1.41, boot 95% CI [0.56, 2.31].

^b^Indirect effect larger for SCS than CS; indirect contrast 1.03, boot 95% CI [0.18, 1.92].

^c^Indirect effect larger for CS than CIS; indirect contrast 1.19, boot 95% CI [0.35, 2.05].

^d^Indirect effect larger for CS than CIS; indirect contrast 0.78, boot 95% CI [0.14, 1.51].

There was a significant association between high scores on SPS+ traits and higher compassion satisfaction (*b* = 1.91, *p* = .033, 95% CI = [0.16, 3.65]) in the analyses described above. Because of this, we re-ran the two mediation models in which full or partial mediation via CS had been demonstrated, but this time the SPS+ score was entered as the predictor and the SPS- score as the covariate. After controlling for SPS- traits, SPS+ provided some protection against burnout and secondary traumatic stress, via its link with increased compassion satisfaction (see Table 2).

## Discussion

It is well-established that individuals with higher levels of SPS experience greater risk of burnout, anxiety, and depression [6,12,44]. The key objective of the present study was to explore the possible mediating roles of perceived COVID-19 impacts, compassion satisfaction, and self-compassion on the relationships between different facets of SPS and measures of burnout, secondary traumatic stress, and negative affectivity in a sample of clinical and counselling psychologists. We demonstrated that SPS- and SPS+ trait clusters play distinct roles in predicting adverse outcomes. The SPS- trait cluster emerged as the predominant predictor of the mediating variables. Thus, when controlling for the SPS+ trait cluster, individuals with higher levels of SPS- traits felt more negatively impacted by the pandemic and reported less self-compassion and compassion satisfaction than those scoring lower on this trait. Each of these variables, in turn, positively mediated multiple adverse outcomes. All three mediators predicted greater risk of burnout; being more negatively impacted by the pandemic and experiencing less compassion satisfaction predicted greater secondary traumatic stress; and being more negatively impacted by the pandemic and reporting lower self-compassion predicted higher levels of stress, anxiety, and depression. We also found that (when controlling for SPS- traits) scoring high on the SPS+ trait cluster offered some protection against burnout and secondary traumatic stress through its positive association with compassion satisfaction. These findings offer insights into some of the factors that were contributing to individual differences in the professional quality of life and mental health of clinical and counselling psychologists during this stressful period.

That the SPS- trait cluster played the strongest role in predicting burnout is consistent with prior work showing that high scores on the ease of excitation and low sensory threshold subscales of the HSPS (which fall in this cluster) predict burnout [6,11,13,45]. The fact that pandemic burden and compassion satisfaction partially mediated this link makes sense, given that these HSPS subscales measure negative emotional reactivity (specifically, the extent to which one is hypersensitive to and easily overwhelmed by stimuli and situations that make one feel uncomfortable). Psychologists and other mental health professionals keenly experienced the burden of the pandemic in their personal and professional lives, without receiving concomitant increases in support in either domain [25–27]. This would have been particularly hard on those who exhibit strong negative emotional reactivity. We also found that low self-compassion partially mediated the link between negative emotional reactivity (as captured by the SPS- score) and increased risk of burnout. This may be because both negative emotional reactivity and self-compassion are associated with low resilience (i.e., difficulty coping during adversity), which is also a predictor of burnout [46,47]. Although resilience was not measured directly in the present study, low resilience may act as a factor contributing to burnout in the counselling and clinical psychologists who endorse strong SPS- traits. This possibility should be explored in future work.

One of the important aspects of our study design was that by entering pandemic burden, compassion satisfaction, and self-compassion as parallel mediators we were able to tease apart the unique contributions these interrelated variables played in mediating adverse outcomes. This proved to be important. For example, past research had suggested that exhibiting low levels of mindfulness, an important aspect of self-compassion) [30,35], is a risk factor for secondary traumatic stress [31]. However, our findings suggest that, in the context of the COVID-19 pandemic, the most important drivers of the link between SPS- traits and secondary traumatic stress in clinical and counselling psychologists were high pandemic burden and low compassion satisfaction. This makes sense as empathetic engagement with clients who had been severely impacted by the pandemic—including those grieving over the loss of friends and loved ones—would have been expected to be traumatizing for therapists [5], as it was for health care providers [48]. We would expect this to be particularly true for those scoring high on SPS- traits, as past work has demonstrated that these traits are a strong predictor of the amount of personal distress people report feeling in response to others’ suffering [20,49].

We found that possessing higher levels of SPS+ traits decreased the risk of burnout and secondary traumatic stress by lowering the perceived burden of the pandemic and increasing compassion satisfaction. These protective effects may relate to the fact that certain components of the SPS+ composite score (e.g., sensitivity to subtle stimuli) are associated with higher resilience via links to mindful attentional awareness [46]. Those scoring high on SPS+ traits are sensitive to nuanced and positively valenced stimuli; they are empathic and “deep” processors [20] who may be able to tolerate challenging situations and manage negative emotions better than those who score low on this trait cluster because they are better able to stay “in the moment” [46]. Indeed, unlike SPS- traits, SPS+ traits predict *lower* levels of personal distress when exposed to tense interpersonal situations or others’ suffering [49]. This could explain why those scoring high (vs. low) on this trait cluster were better able to deal with pandemic-related burdens and got more satisfaction from doing their jobs—both of which helped to reduce their risk of burnout and secondary traumatic stress.

Our investigation also focused on stress, anxiety, and depression in the clinical and counselling psychologists who comprised our sample. Here, we observed that feeling the negative impact of the pandemic more keenly and reporting lower levels of self-compassion explained the relationship between SPS- traits and these indicators of negative affectivity. The relationship between SPS and self-compassion has received little attention to date. However, stronger expression of SPS- traits (as reflected in low scores on the ease of excitation and low sensory threshold subscales of the HSPS) has been associated with low levels of mindfulness, which is a key component of self-compassion [30,35]. Past work also supports the view that scoring low on self-compassion predicts higher levels of stress, anxiety, and depression in therapists [50]. Working to become more aware of their current situation and learning to embrace their thoughts and emotions without judgement may make it easier for psychologists and counsellors scoring high on SPS- traits to tolerate negative emotions, self-regulate, and act with self-compassion instead of self-criticism, improving their overall mental health and well-being. Improving mindfulness and becoming more self-compassionate does not happen overnight; therefore, it is important for these qualities to be practiced now to protect highly sensitive psychologists and counsellors during and in the aftermath of future stressful events. This is critical as these events can have long-lasting impacts. For example, research conducted after prior epidemics (i.e., severe acute respiratory syndrome and Middle East respiratory syndrome) identified ongoing high rates of anxiety and depression up to three years after these outbreaks [51,52]. Taking this kind of proactive approach and using research such as this to inform other systems-level changes will help protect workers in the future [4].

### Limitations and future directions

As noted above, after exclusions our final sample of 95 participants was slightly smaller than that recommended by our a priori power analysis. Given this, further research with a larger sample size is recommended to assess the generalizability of the findings. This work should also take into account how variables related to participant demographics and work history impact the results. Unfortunately, due to a technical issue, data related to these variables were not collected in the present study. Other recent findings suggest that they are important to consider. For example, years in practice has been positively associated with the perceived burden of ensuring compliance with guidelines designed to limit the spread of COVID-19 [26] and with burnout and secondary traumatic stress [27]. Interestingly, although research carried out with university samples during the pandemic suggested that females score higher on both SPS- and SPS+ traits and felt more negatively impacted by the pandemic than males [20,53], SPS proved to be a more important predictor of anxiety and depression than sex or COVID-19 impacts [53].

The current study utilized a cross-sectional study design and regression-based analyses; as such, causal connections between the variables cannot be made. Interestingly, findings from a recent longitudinal study that included a cross-lagged path analysis revealed a *bidirectional* relationship between emotion dysregulation and burnout in mental health professionals over a 10-month period during the pandemic, with each predicting increases in the other [54]. A longitudinal study design would be needed to determine if causal relationships exist between our study variables, and if any of the relationships between our variables are also bidirectional.

Future research should explore ways to prevent or reduce burnout and negative affectivity, particularly in high-risk groups such as those displaying strong SPS- traits. Our findings suggest that recognizing that one is highly sensitive—and learning more about the role that SPS- traits (in particular) play in the workplace—would be important first steps in being able to implement proactive strategies to improve one’s professional quality of life and well-being. Taking steps to become more mindful and self-compassionate should help in this regard [31]. Indeed, mindfulness training has been shown to improve resilience and reduce burnout in highly sensitive individuals by lowering levels of emotional reactivity [46]. Other forms of psychotherapy (e.g., cognitive behavioural therapy, dialectical behaviour therapy) and psychoeducation regarding coping and self-care strategies (such as avoiding overstimulation and setting boundaries) may also prove useful. Methods of reducing burnout at an organizational level include providing professionals with a level of influence over procedures, reducing the number of administrative duties, and increasing support provided by supervisors [5,55]. Additionally, reducing the total number of hours worked or caseload can also be helpful strategies to reduce burnout, as these variables are directly related to burnout [5,55].

## Conclusion

The relationship between SPS and increased risk of poor professional quality of life has been explored in a number of studies focused on a range of helping professionals. Our findings provide a more nuanced look at factors underlying this relationship. We focused on psychologists and counsellors working in the midst of a global health emergency. By identifying risk and protective factors for adverse outcomes and their possible mechanisms of action, this research may inform the development of proactive interventions designed to prevent burnout, secondary traumatic stress, and negative affectivity in highly sensitive individuals who perform the vital work of providing mental health services during future stressful world events.
